# Applying the WHO International Classification of Functioning, Disability and Health in Nursing Assessment of Population Health

**DOI:** 10.3390/ijerph15102245

**Published:** 2018-10-13

**Authors:** Juan Gómez-Salgado, Lia Jacobsohn, Fátima Frade, Macarena Romero-Martin, Carlos Ruiz-Frutos

**Affiliations:** 1 Department of Nursing, University of Huelva, 21007 Huelva, Spain; jgsalgad@gmail.com; 2 Safety and Health Posgrade Program, Universidad Espíritu Santo, Samborondón, Guayaquil 091650, Ecuador; 3 Atlântica Health School, 2730036 Barcarena, Portugal; ljacobsohn@uatlantica.pt (L.J.); ffrade@uatlantica.pt (F.F.); 4 Centro de Medicina de Reabilitação do Alcoitão, 2649506 Alcabideche, Portugal; 5 Centro Universitario de Enfermería Cruz Roja, Universidad de Sevilla, 41009 Sevilla, Spain; 6 Department of Sociology, Social Work and Public Health, Universidad de Huelva, 21007 Huelva, Spain; frutos@uhu.es

**Keywords:** classification, international classification of functioning, nursing, disability and health, quality

## Abstract

Classification systems constitute an important contribution to nursing practice, as they provide standardized frameworks for communication between nurses and other healthcare professionals. International Classification of Functioning, Disability and Health (ICF) provides a unified and standardized language, as well as a working structure, for the description of health and health-related states. This paper aims to describe some of the available classifications used in nursing practice and to identify the potential value provided by the application of the World Health Organization (WHO) International Classification of Functioning, Disability and Health by all healthcare professionals. With this purpose, a concept analysis was conducted. The relevant nursing classifications were analyzed and related evidence on the use of ICF classification was reviewed to provide a discussion on the application of ICF in nursing practice. The use of ICF could be beneficial in different areas of nursing practice, as it provides a more comprehensive framework to classify nursing outcomes and interventions, improving areas such as interprofessional communication and optimization of care. Although there is published evidence on the use of ICF core sets, further research is needed on this area.

## 1. Introduction

Classification systems constitute an important contribution to nursing practice, as they provide standardized frameworks for communication between nurses and other healthcare professionals. They can also be used as a guide to choose the best interventions and as a framework for recording them and assessing outcomes, thus providing higher quality care. There are different nursing classification systems, such as the North American Nursing Diagnosis Association classification (NANDA), the Nursing Interventions Classification (NIC), the Nursing Outcomes Classification (NOC), the International Classification for Nursing Practice (ICNP), and the International Classification of Functioning, Disability and Health (ICF).

In 2001, the World Health Organization (WHO) created a multidimensional and interactive classification for universal use, with the aim of providing a unified and standardized language, as well as a working structure for the description of health and health-related states—the International Classification of Functioning, Disability and Health (ICF). This classification introduced a shift from the bio-medical paradigm towards a biopsychosocial and comprehensive model based on functionality and human disability [1]. Previous WHO classifications assumed that disability began when health ended. However, the ICF favors measuring the performance of an individual in society, regardless of the factors defining their disability or limitation. This entails a drastic change of approach, as instead of focusing on the disability of individuals, their functioning levels and potential are valued. For this reason, the ICF is a much more versatile tool with a wider range of applications in comparison with more traditional classifications of health and disability. This new classification plays a key role not only in assessments and interventions related to patients’ functional parameters but more significantly in the definition, planning, measurement, and evaluation of policies, services and resources. This can apply not only to healthcare environments but also to other sectors with a view to expanding social policies directly or indirectly related to human functioning and disability [1]. 

The use of classification systems in nursing has made significant contributions to clinical practice, as they provide nursing professionals with a standardized language. It has also improved communication between nurses and other healthcare professionals and it has increased the quality of data recording and organization. In addition to this, it has enabled a more rigorous assessment of nursing care, thus allowing more effective interventions. These advantages become apparent in an improvement of the care provided to patients [2]. Nursing classification and standardized nursing languages have shown major benefits for nursing documentation and research. They are presented as reliable methods to record, organize, and analyze nursing information that is gathered in electronic healthcare records, which facilitates data compiling for nursing research [3]. In short, the use of a standardized terminology improves communication between nurses and other team members, enhances continuity of care, facilitates care and its documentation, and makes nursing practices visible [4]. 

ICF is an international classification proposed by the WHO to assess health-related states and functionability. Although it is a multidisciplinary tool, it was not designed to be used by nursing professionals exclusively, as other nursing classifications. It would be interesting to analyze ICF from the nursing approach and to compare it with other genuine nursing classifications in order to assess and prove its applicability in the nursing field.

This article aims to describe some of the available classifications used in nursing practice and to identify the potential value provided by the application of the WHO International Classification of Functioning, Disability and Health by all healthcare professionals. This could imply qualitative and quantitative improvements of nursing care in terms of quality, safety, and cost-effectiveness.

## 2. Materials and Methods 

A concept analysis was conducted according to the Morse concepts comparison methodology. The Morse model explores the concepts based on the level of development/maturity revealed by theirinternal structures, on their representativeness, and in their relationship with other concepts [5]. By using the Morse model, we analyzed the preconditions of the following different concepts: ICF, NANDA, NIC, NOC, and ICPN. Then, we described the nursing responses to the different concepts under study. Finally, we obtained the results, which explained how nurses use the different concepts and the also outcomesfrom this usage. Thus, scientific literature about the different concepts (ICF, NANDA, NIC, NOC, and ICPN) was consulted, with the aim to identify each of their objectives, structures, and categories, and also to consider the applicability and possible benefits of each of these concepts in nursing practice.

The relevant nursing classifications were analyzed and compared. Evidence on the use of ICF classification was reviewed to provide a discussion on the application of ICF in nursing practice.

## 3. Results

Nursing classifications share similarities, although each one has certain particularities. All of them are used internationally, but only the ICF focuses on interdisciplinary use, as shown in Table 1. 

The ICF core sets have been developed to facilitate the application of the ICF framework. They are lists of categories that have been developed by expert nurses’ consensus on scientific evidence to classify relevant performance characteristics of patients in specific contexts or with specific health conditions [6]. The ICF can be extended for application in specific situations, such as acute hospital situations [7]. It could also be a highly useful tool in public health, since it describes the functionality of individuals and populations from a biopsychosocial perspective, avoiding the reductionism of diagnosis and complementing indicators that are generally used to measure mortality or morbidity by using an international and standardized language. This language describes and classifies health and its related dimensions to promote the use of a common framework for measuring outcomes [8]. The ICF can also be useful in evaluation and decision-making processes in the clinical practice of different specialties, as it could facilitate communication not only among members of the multidisciplinary team but especially between patients and healthcare professionals [9,10].

Nursing professionals rely on the NANDA taxonomy to make nursing diagnoses, on the NIC to describe nursing interventions, on the NOC to describe nursing outcomes, and on the International Classification for Nursing Practice (ICNP) to describe diagnosis, nursing interventions, and interventions outcomes [11]. There are few studies that establish the criteria for the classification of nursing diagnoses and how these classifications relate to the criteria. Various authors have identified and assessed how these criteria are linked to the ICF, the ICNP, and the NANDA, and suggested that, in the light of the studies found and considering the opinions of expert nursing professionals, although the three classifications use diagnostic terms in nursing, NANDA was the recommended one for clinical practice and for electronic documentation in nursing [12].

A nursing diagnosis is a clinical judgment about the response of an individual, a family, or a community to actual or potential health problems and life events. Nursing diagnoses provide basic information to define nursing interventions and to achieve outcomes for which nursing professionals are responsible. The NANDA taxonomy, in its eleventh edition (NANDA 2018–2020), includes 244 nursing diagnoses. Nursing diagnoses are classified within a class (e.g., class 2—health control), and this class is part of a wider domain (e.g., domain 1—health promotion). The term domain refers to an area of activity, study, or interest. Class refers to the classification of people or elements by quality, group, or degree [13]. The NANDA classification system has been widely disseminated and applied worldwide. Nursing diagnoses originate from clinical judgements and consider the characteristics or reactions of individuals, families, or communities to health problems that can be observed and verified. These characteristics or reactions operate as evidence or as implications of a disease or a state of health and well-being, and they can be used as the basis for nursing interventions [14].

The NIC system, which includes nursing interventions, is based on the range of activities that describe the professional actions performed in those interventions [14]. Along with the need for the creation of a taxonomy for nursing diagnoses (NANDA) arises the need for developing a vocabulary and classification of nursing interventions by means of the NIC taxonomy, and the creation of vocabulary and a classification of nursing outcomes with the use of the NOC taxonomy. As in the NANDA classification system, the concepts described in the NIC (e.g., field: family; e.g., class: intra-partum and postpartum care) and the NOC (e.g.,domain: functional health; e.g., class: mobility) are also classified in greater domains, each of which includes different classes. The NIC focuses on nursing behaviors, i.e., actions that contribute to nursing care given to a patient, group, or community and that help nurses achieve the expected results. The NIC can be used in the different areas where nursing care is provided (intensive care unit, primary care, home care, palliative care, etc.) and in all nursing specialties (nursing rehabilitation, psychiatric and mental health nursing, medical-surgical nursing, etc.). The NOC is a comprehensive and standardized classification of expected patient outcomes that can be used to assess the results and effectiveness of nursing interventions [15].

Another classification currently used by nursing professionals is the ICNP, which includes diagnoses, interventions, and nursing outcomes [16]. This system combines terminology into a structure with different axes (i.e., one or several simple concepts are combined to establish complex concepts, providing a clear meaning of nursing diagnoses, interventions, or outcomes) [2]. The ICNP has emerged from the need for a clear structure for nursing practice. This prompted the creation of a terminology that was developed using modern scientific technology and that included global participation towards its development and its application into clinical practice. The use of this terminology generates data that can be collected systematically to analyze the clinical environment, the existing resources for nursing practice, the nursing care provided, and patient outcomes. When data collection methods reflect a range of healthcare environments, they are progressively and carefully developed and implemented, allowing nurses to make comparisons and to evolve towards the safe provision of care at every level, including local and international environments [17,18]. The main objectives of the ICNP are as follows: to establish a common language to describe nursing practice in order to improve communication among nurses and between them and other healthcare professionals; to describe the nursing care provided to individuals, families, and communities in different contexts; to allow the comparison of data in nursing records for different populations, clinical situations, geographical areas, and time periods; and to promote research by providing access to the available data in nursing-specific information systems and other healthcare information systems, thereby facilitating the gathering, dissemination, and analysis of evidence on nursing practice, which could subsequently lead to the development of healthcare policies [17]. In order to make ICNP more rigorous, some changes have taken place since its creation. The first version used was the beta version and the current one is version 2, which presents a framework including seven axes (focus, judgment, client, action, means, location, and time) that nurses can use to make diagnoses, perform interventions, and consider nursing outcomes. The ICNP axes used for the formulation of diagnoses, interventions, and nursing outcomes are as follows [17]:

1. Focus, which refers to the relevant area of nursing care.
2. Judgement, which is the decision or clinical opinion for nursing practice.
3. Means, which refers to the way or method of carrying out an intervention.
4. Action, which is the intentional process applied to or carried out by a client.
5. Time, which refers to the moment, time interval, or duration of a situation.
6. Location, which refers to the anatomical area or physical context of a diagnosis or intervention.
7. Client, who is the recipient of nursing diagnoses and interventions.

## 4. Discussion

An analysis of the evidence of the application of ICF in nursing practice provides several recommendations, and the ICF stands out for having the potential to help nurses consider the cultural, social, and political dimensions of disability in clinical practice.Although this classification is not specifically designed for nurses, the ICF is relevant for nursing care. The lack of consensus can be overcome by including nurses as participants in future review studies.The ICF may be a useful tool for nurses to classify and report aspects of the functional status of patients—the three-digit level of categories has proved to be suitable for this purpose. The use of this classification should be encouraged by the nursing community due to its relevance and its potential multidisciplinary use in patient care. The ICF classification is relevant for nursing assessment, optimization of patient care, and management of resources for different levels of neurological, cardiopulmonary, and musculoskeletal intensive rehabilitations. 

In this sense, the ICF covers the aims of nursing interventions, and it also applies in acute and post-acute rehabilitation, as these interventions may be associated with categories, and this method can be useful in clinical practice. Initial issues in the adaptation of the terminology to nursing entail that the ICF working structure can be adjusted, but more evidence is needed to support these results. Furthermore, the ICF core sets for acute and post-acute hospital rehabilitation are relevant for rehabilitation nursing, as their association with nursing interventions can be used to describe intervention objectives. This can improve communication among professionals, while respecting patient needs. ICF core sets for rheumatoid arthritis have been specifically validated for nursing, and the results showed a wide application in clinical practice. For example, there are valid and predictive ICF core sets for the use of the Barthel index, and its incorporation into decision making in nursing management could lead to a significant improvement in practice, as it involves providing staff with further training.

The existence of different terminologies used in nursing practice prompted the creation of a reference model to represent nursing concepts that could also be integrated into other healthcare disciplines. The NANDA, NIC, NOC, and ICNP classification systems have been associated with their taxonomic code, thus enabling the use of classification systems in healthcare computer software [17]. The ISO 18104: 2003 standard is used to establish a semantic equivalence between NANDA-I taxonomy diagnoses and the ICNP language system [19]. This standardizes nursing language, as it brings together the different classification systems in order to promote the integration of information systems and the possibility of comparing nursing terms with those in other healthcare terminologies. It also establishes criteria to evaluate existing classification systems and it allows revisions in their standardization. International standardization facilitates the creation of a reference model for two key nursing concepts: diagnoses and interventions. For instance, this standardization establishes that the use of ICNP nursing diagnoses should include the focus and the judgment axes [20].

Some of these taxonomies, such as NANDA, NOC, or NIC, complement each other and should be used in conjunction with each other in order to cover every phase of the nursing process (assessment, diagnosis, planning, implementation, and evaluation). NANDA refers to nursing diagnosis (diagnosis), NOC refers to nursing outcomes (planning and evaluation), and NIC refers to nursing interventions (planning and implementing) [21]. However, as both NANDA and ICNP deal with nursing diagnosis, they cannot be used together, as they label the same concepts [17]. In addition, the ICF can be used as a complement to other taxonomiesduring the assessment phase of the nursing process [1].

It is important to point out that although the ICF classification is not aimed to formulate diagnoses, it is a universal classification that can be used by different healthcare professionals to describe functioning status in comparison with disability. For instance, the approach used in rehabilitation follows a continuous and cyclical process [22]. All healthcare professionals involved in this process, including nurses, require an effective system for managing and for assessing functionality in order to achieve the aims set by multidisciplinary teams [23,24].

In this sense, the ICF is a relevant tool for nurses involved in rehabilitation, as it can be used as a framework for research, training, and clinical practice. On the other hand, it has the potential to provide a rationale for clinical practice, when considering the cultural, social, and political dimensions of disability [25]. Two of the more recent studies on the application of ICF in nursing practice [26,27] have established it as a potentially useful framework to describe the functionality of specific nursing aims and nursing elements, as this classification can be used by all healthcare professionals to standardize the documentation of patient care.

Although there is published evidence on the use of ICF core sets, further research is needed in this area. Nursing research on ICF is not widespread and, currently, it does not seem to be appealing to professionals. This presents an issue when trying to encourage nurses to use this classification—a notable problem, given that its use has been decreasing in the last decade. However, publications about the ICF core sets have steadily increased, including ICF core sets for multiple sclerosis, spinal cord or vertebral injury, head injury, hand conditions, musculoskeletal, cardio-respiratory and neurological conditions in hospital, inflammatory bowel disease, head and neck cancer, and acute and post-acute conditions. These studies are essential in order to facilitate ICF implementation in clinical practice by healthcare professionals in general and, particularly, by nurses. Validation studies of core sets carried out by nurses are needed to promote greater autonomy in relation to the classification, implementation, and management of patient functioning status.

Conducting research on nursing classifications helps us to understand their application on practice and allowsus to assess the outcomes of nursing diagnoses [28]. However, it is important to understand the benefits of using a more comprehensive and multidisciplinary classification to enhance the knowledge gained by applying nursing classifications.

Strengths of this article include providing a comparison and discussion of the different nursing classification systems and establishing recommendations for the use of the WHO ICF in practice based on the relevant evidence. A limitation of the discussion provided is that this is based on a concept analysis, and as such, further information on the application of recommendations in specific environments must be collected from applied research.

## 5. Conclusions

Different nursing classification systems are available for professionals. Their use enables the implementation of systematic and standardized practice, thereby improving quality of care. The WHO ICF could be usefully applied in nursing practice to improve communication among the different members of multidisciplinary teams, as it provides a comprehensive approach to health and health-related states by considering the performance of individuals in society in their different dimensions. The use of ICF would provide a common language for health professionals when dealing with patients’ functionality, so it will improve communication and patient monitoring.

Although there is a long way to go, the use of ICF in nursing practice could promote the further development of a multidisciplinary approach to delivering quality healthcare while also reducing the barriers that nurses encounter in their everyday work.

## Figures and Tables

**Table 1:** Nursing classification comparison.

Classification System	Year	Objectives	Structure	Categories
ICF* (WHO) †	2013	Evaluation of disability in various scientific contexts, clinical, administrative and social policy; Establish a common and universal language; Providing an encoding scheme.	- Components - Health Domains - Constructs - Positive Aspects - Negative Aspects	The maximum number of codes available is 34 at chapter level and 362 at the second level. At third and fourth levels, there are up to 1424 available codes that together constitute the full version of the classification.
**NANDA**‡	2017	Establishing nursing diagnoses; Providing a universal language; Classifying diagnoses taking into account domains and classes.	- Domains - Classes	244 diagnosis classified in 13 domains and 47 classes (from 2 to 6 classes in each domain).
**NOC**§	2016	Identifying nursing outcomes and evaluating nursing interventions and outcomes; Establishing a link between NANDA nursing diagnoses and nursing interventions of NIC; Classifying nursing results taking into account fields and classes.	- Domain - Class - Outcome - Indicators	The categories include 6 domains, 24 classes, and 490 nursing outcomes.
**NIC**||	2016	Establishing nursing interventions/activities and planning to develop and implement these interventions; Acting as a liaison with the nursing diagnoses of NANDA; Classifying nursing interventions taking into account fields and classes.	- Domainv - Class - Intervention - Activities	The categories include 7 domains, 30 classes, and 554 nursing interventions.
**ICPN**¶	2016	Identifying nursing diagnoses; Prescribing nursing interventions; Evaluating nursing outcomes; Providing a universal language for nursing practice; Classifying nursing phenomena into axes; Enabling the comparison of data in medical records; Promoting nursing research.	7 axes: - Focus - Judgment - Resources - Action - Time - Location - Client	Nursing diagnoses, interventions, and outcomes are the result of mergingthe different axes and the number of concepts that each axis integrates.

* International Classification of Functioning, Disability and Health; † World Health Organization; ‡ North American Nursing Diagnosis Association; §Nursing Outcomes Classification; || Nursing Interventions Classification; ¶ International Classification for Nursing Practice.
